# Stroke Caregiver Education and Training in an Inpatient Sub‐Acute Setting: A Retrospective Clinical Audit

**DOI:** 10.1111/jep.70690

**Published:** 2026-10-20

**Authors:** Madelaine McCarthy, Nicky Baker, Natasha A. Selenitsch, Maayken van den Berg

**Affiliations:** ^1^ College of Health and Enablement Flinders University Adelaide South Australia Australia; ^2^ Department of Physiotherapy Barwon Health Geelong Victoria Australia; ^3^ Caring Futures Institute, College of Health and Enablement Flinders University Adelaide South Australia Australia

**Keywords:** caregiver, education, multidisciplinary, rehabilitation, stroke, training

## Abstract

Caregiver burden is a significant issue post‐stroke. Caregivers demonstrate unmet needs throughout the rehabilitation phase, with limited literature that delves into the details of caregiver education and training (CGET) provision prior to discharge.

**Objectives:**

To identify current practice of multidisciplinary team documentation of CGET following stroke on an inpatient subacute neurosciences ward.

**Methods:**

A retrospective clinical audit was conducted on 109 stroke admissions on an inpatient subacute neurosciences ward. Documented demographic information, admission and discharge Functional Independence Measure scores, and CGET from the multidisciplinary team were collected using a purpose‐built audit tool. Descriptive analysis and sub‐group comparison explored the findings against the Stroke Guideline recommendations.

**Results:**

Data from 109 records were included, which identified 1398 episodes of CGET. Social work (SW) (79.9%) and occupational therapy (OT) (77.1%) documented CGET more consistently than other disciplines. CGET was significantly more likely to be documented for patients with cognitive impairment, yet only three of 28 (10.7%) individuals with memory impairment had documented CGET relating to memory strategies. Nursing staff documented minimal CGET across all domains, including management of continence.

**Conclusions:**

Inconsistent CGET was documented for stroke recovery relating to activities of daily living, mobility and cognition. Variable documentation practices highlighted challenges with interpretation. Further research is needed to determine whether functional limitations of patients are being matched with the appropriate CGET.

**Practice Implications:**

multidisciplinary team caregiver education and training provision and documentation needs to be consistent, particularly relating to cognition, continence, mobility and activities of daily living.

## 1 Introduction

Stroke is the second leading cause of death and the third leading cause of disability worldwide. Whilst the prevalence of stroke has decreased over time, the overall burden of stroke has remained high with a significant economic impact globally [1]. Over 70% of patients who survive the acute phase of stroke will endure some degree of persistent physical or cognitive disability. Many of them will require ongoing caregiver support to engage in life at maximum capacity [2]. Because stroke is so sudden, families and supporters are thrown into a caregiver role without warning [3]. This can be overwhelming emotionally and physically, leading to significant stress for both patients and caregivers [4]. For the purpose of this study, caregivers are defined as ‘unpaid caregivers including spouse or partner, family members, friends or significant others who provide physical and emotional support to stroke survivors’ [5].

Caregivers frequently feel under‐prepared for the patient's discharge home post stroke [6]. Unmet needs for caregivers include education and training about stroke, coping with their own and the loved one's emotions, and accessing services [6]. Caregiver education is a process of sharing information to increase knowledge, attitude and skills. Caregiver training can be considered the provision of practical opportunities for the caregiver to observe, participate, problem solve and demonstrate skills, and be provided with feedback [3]. There is substantial overlap between these two terms in the literature, making it difficult to decipher what occurs in clinical practice.

A large proportion of patients and caregivers are dissatisfied with the information provided in rehabilitation [7] and some may prefer more practical experiences [8]. Caregivers can feel excluded during the rehabilitation process. Training to prepare them for discharge home can be inconsistent [9]. Ongoing difficulties with caregiver duties at home can increase rates of depression, low mood and overall reduced quality of life in both caregivers and the patient [10].

Caregiver education and training (CGET) post‐stroke can improve patient and caregiver outcomes such as quality of life and mood [11], caregiver preparedness for discharge [12], satisfaction, quality of life, and reduce patient anxiety and depression [13]. In inpatient rehabilitation settings, caregiver involvement can increase patient motivation for recovery, mood and self‐efficacy that can improve patient participation and functional outcomes [14].

Various national guidelines recommend CGET as paramount in preparing the stroke patient and their caregivers for discharge home [15, 16]. However, differences exist regarding the type, specificity and quantity of CGET, impacting clinicians' ability to apply the guidelines accurately in clinical practice [17]. Application of guidelines is exacerbated by variability in stroke presentation and differences in functional deficits, which challenge consistent provision of CGET [18]. Nonetheless, CGET relating to the patient's functional impairments is crucial for a seamless transition home.

The Australian Stroke Rehabilitation Audit in 2020 showed that 84% of health services documented CGET and 63% of caregivers were provided with written information on stroke during the patient's admission [19]; however, the audit did not detail the types of CGET or clinical disciplines involved in provision of CGET. The multidisciplinary team (MDT) is integral to preparing the patient and caregiver for discharge; however, few studies explore the detail of CGET throughout the rehabilitation admission. Methods of caregiver training have been explored [20] but there is limited evidence of CGET documented by health professionals in a subacute setting, particularly in relation to the patient's functional needs. Therefore, the aims of this study were to identify current practice of stroke CGET documented by the MDT in an inpatient sub‐acute ward; to determine details of CGET for each discipline; and to compare against the Australian Stroke Guidelines.

## 2 Methods

This retrospective audit reviewed clinical records for episodes of CGET documented by the MDT, in adults admitted to an inpatient, sub‐acute neurological rehabilitation ward in a large regional city in Victoria, Australia (Table 1, Inclusion and exclusion criteria). This ward provides rehabilitation for acquired brain conditions such as stroke and brain tumours, as well as progressive neurological conditions like multiple sclerosis. The focus of this audit was specific to education and training for rehabilitation of stroke. Records from 2020 were excluded due to COVID‐19 changing admission, treatment and working conditions that could skew the data.

**Table 1:** Inclusion and exclusion criteria.

Inclusion criteria	Exclusion criteria
Adults ≥ 18 years of age. Admission to Barwon Health McKellar Centre neurological rehabilitation ward Discharge date occurred between 01/01/2019 to 31/12/2019 inclusive Acute stroke diagnosis on admission (haemorrhagic or ischaemic) Identified caregiver(s) during admission	Other neurological disorder not diagnosed as an acute stroke event Admission length of stay 2 days or less Unplanned interruption to rehabilitation programme for entirety of admission Patient passed away or became palliative

Ethics approval was obtained through the Barwon Health Human Research Ethics Committee (approval number 21/177). Data were deidentified prior to analysis.

An *a priori* audit tool was purposefully developed to extract (1) Demographic, (2) CGET (3) admission Functional Independence Measure (FIM) [21] and (4) discharge FIM data. The change in FIM measured change in functional ability. The audit tool was developed, trialled, and revised with the research team for the data collection process. Documented evidence of CGET was collected from the clinical records. Caregiver ‘education’ was interpreted as provision of verbal or written information; whereas ‘training’ was interpreted as a practical demonstration or hands‐on skills practice [3]. For initial coding, the first five medical records (5%) were cross checked by a second investigator to gain agreement on coding ‘education’ or ‘training’. Discrepancies in coding were resolved by returning to the medical records to identify where and how CGET was recorded so that coding agreement could be reached by consensus. Codes were generated based on type of CGET and merged into common topics at the conclusion of data collection. Family meetings were recorded separately due to difficulty interpreting which disciplines provided what elements of education from the documentation. All data were collected by the Primary Investigator.

Deidentified data were summarised using descriptive statistics (means and standard deviations for scale data; frequency and proportions for categorical data). Subgroup analysis using Chi‐Square tests of Independence determined which patient characteristics were associated with CGET. Variables of interest included patient age, presence of a nominated caregiver, discharge destination, FIM discharge score including cognition and mobility. Rationale for these subgroups were determined based on the literature regarding differences in rehabilitation experiences based on patient age [22] and caregiver identified for discharge [19]. Data were also grouped according to discharge destination (home or other) to consider CGET for level of caregiver involvement for home discharge, and patient level of function on discharge. Statistical significance was set at *p* ≤ 0.05. All analysis was conducted in SPSS version 28. Data for each discipline were further aligned with the National Stroke Foundation rehabilitation guidelines, where each code was allocated a sub‐component within the three domains of primary rehabilitation; managing complications and discharge planning; and transfer of care & community integration.

## 3 Results

Data were included from the 109 eligible records within the timeframe. These identified 1398 episodes of CGET. Most patients were older adults (71.0 ± 13.8 years), had suffered an ischaemic stroke (84.4%) and were discharged home (72.5%) (Table 2, Demographic Characteristics).

**Table 2:** Patient demographic characteristics.

Patient characteristics (*n* = 109)	*n* (%)
Age, years	
Mean (range)	71.0 (31–94)
Gender	
Male	56 (51.4)
Female	53 (48.6)
Diagnosis	
Ischaemic	92 (84.4)
Haemorrhagic	17 (15.6)
Length of stay, days	
Mean (min–max)	33.0 (4–113)
FIM admission score	
Mean (SD)	68.0 (21.2)
Pre‐morbid FIM walk/locomotion	
6 or 7 (independent with/without equipment)	103 (94.5)
1–5 (second person required)	6 (5.5)
FIM discharge score	
Mean (SD)	95.4 (25.2)
Discharge destination	
Home	79 (72.5)
Residential aged care	11 (10.1)
TCP‐R	7 (6.4)
Regional/rural transfer^a^	7 (6.4)
Acute transfer^b^	5 (4.6)
Number of caregivers identified	
1	35 (32.1)
2	36 (33.0)
*≥ *3	38 (34.9)

Abbreviation: TCP‐R, transitional care programme (residential).

^a^ Inpatient hospital transfer closer to discharge destination.

^b^ Returned to acute hospital secondary to medically unwell.

Social work (SW) (79.9%) and occupational therapy (OT) (77.1%) documented the most CGET (Figure 1, Percentage of included records that documented multidisciplinary CGET). Family meetings occurred in 32.1% (*n* = 35) admissions. Additional community stroke supports were documented for 1.9% caregivers and a further 11.4% caregivers for additional caregiver supports or allowances.

**Figure 1:**
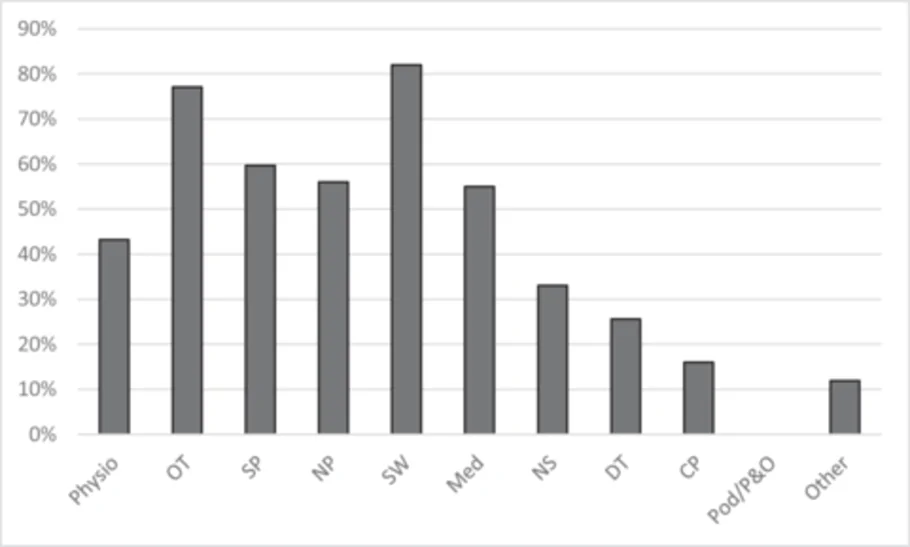
Percentage of included records that documented multidisciplinary CGET. *'Other' disciplines included pharmacy, diabetes education and speciality discharge destinations providing education. **Physio = physiotherapy, CP = clinical psychology, DT = dietitian, NS = nursing staff, NP = neuropsychology, OT = occupational therapy, Pod/P&O = Podiatrist/Prosthetics & Orthotics, SP = speech pathology, SW = social work.

Documentation of discipline‐specific CGET varied. Of the 109 OT documented occasions of CGET, most were for equipment needs (*n* = 51, 46.6%), recommendations on activities of daily living (ADL) (*n* = 48, 44.0%), and home modifications (*n* = 41, 37.6%). Social work (SW) CGET (*n* = 105) related to community services for home discharge (*n* = 44, 41.9%) or other discharge options (*n* = 45, 42.9%), as well as finances (*n* = 24, 22.9%) and legal support (*n* = 15, 14.3%). Physiotherapy (PT) CGET was mainly mobility education (*n* = 27, 24.8%), as well as physical training with caregivers on mobility and transfers (*n* = 17, 15.6%) particularly car transfers (*n* = 12, 11.0%). Speech pathology (SP) CGET (*n* = 72) documented education on communication strategies (*n* = 12, 16.7%), preparation of modified diets or fluids (*n* = 13, 18.1%) and swallowing strategies (*n* = 9, 12.5%). Dietetics (DN) CGET (*n* = 66) was for diet and/or fluid recommendations (*n* = 15, 22.7%), while clinical psychology (CP) (*n* = 25) documented education on strategies to improve mood/anxiety (*n* = 2, 8%). There were no episodes of CGET documented by a podiatrist and/or prosthetist/orthotist.

For patients discharged home and requiring assistance from another person to walk and/or transfer (interpreted as FIM score ≤ 5), physical training was documented by OT/PT on the ward or at home for 11 (63.6%) patients. Mobility education rather than training was documented by PT/OT for 90.9% of these occasions. For patients with a discharge FIM score of 5 or less for transfers or walking/locomotion, PT was statistically more likely to document CGET (*X* ^2^ = 16.29; *p* < 0.001), as were SW (*X* ^2^ = 4.82, *p* = 0.03), medical (*X* ^2^ = 13.76, *p* < 0.001) and nursing (*X* ^2^ = 17.21, *p* < 0.001). Inpatient or home exercise programmes CGET were documented by PT in 2.8% and OT in 14.8% clinical records.

Seven patients were discharged requiring physical assistance with showering and/or toileting but only one caregiver had documented evidence of personal hygiene training by an OT. Of the 28 patients discharged home requiring a level of assistance for memory (FIM score 4 or less), only 3 (10.7%) had documented evidence of CGET; one (3.6%) from OT and two (7.1%) from neuropsychology. Eight (7.3%) patients were discharged home with assistance required for bowel and/or bladder management. Nursing staff documented CGET for indwelling catheter management for two patients; however, there was no documentation of any CGET relating to continence aids or management.

Older patient age was significantly associated with CGET by OTs (*X* ^2^ = 4.72, *p* = 0.03) but not by other disciplines (Table 3, Subgroup analysis of records with documented CGET by discipline). Patients discharged to a different setting than home had significantly more CGET documentation from medical (*X* ^2^ = 5.60, *p* = 0.02) and nursing (*X* ^2^ = 5.39, *p* = 0.02) clinicians. Conversely, dietetics were more likely to document CGET for discharge home compared to another destination (*X* ^2^ = 7.1, *p* = 0.01). For those discharged home with a total FIM score of 100 or less, indicating some level of assistance required for daily activities, CGET was significantly more likely to be documented by PT (*X* ^2^ = 22.97, *p* < 0.001), SW (*X* ^2^ = 5.69, *p* = 0.02), medical (*X* ^2^ = 20.17, *p* < 0.001), nursing (*X* ^2^ = 14.08, *p* < 0.001) and dietetics (*X* ^2^ = 3.88, *p* = 0.05).

**Table 3:** Subgroup analysis of records with documented CGET by discipline.

	PT	OT	SP	SW	MED	NS	DT	CP	NP
Age (years)									
< 65	16	24	18	27	21	16	4	4	7
≥ 65	31	60	25	59	39	20	13	0	7
*X* ^2^, Phi	0.00, 0.00	**4.72, 0.21** *	0.87, −0.011	0.21, 0.05	0.07, −0.30	2.64, −0.16	0.73, 0.11	#	0.05, −0.05
Presence of a caregiver									
No Caregiver	23	40	22	45	30	17	9	2	5
Caregiver	24	44	21	41	30	19	8	2	9
*X* ^2^, Phi	0.08, 0.03	1.18, 0.10	0.06, −0.03	0.65, −0.08	0.01, 0.01	0.23, 0.05	0.02, −0.02	#	#
Discharge destination									
Home	30	64	32	58	38	21	16	4	9
Other	17	20	11	28	22	15	1	0	5
*X* ^2^, Phi	3.10, 0.17	2.53, −0.15	0.66, −0.10	3.70, 0.19	**5.60, 0.23** *	**5.39, 0.22** *	**7.10, −0.33** *	#	#
FIM discharge scores									
≤ 100	33	39	22	44	38	25	12	0	6
> 100	14	45	21	42	22	11	5	4	8
X^2^, Phi	**22.97, −0.50** **	0.85, −0.09	0.06, −0.03	**5.69, −0.23** *	**20.17, −0.46** **	**14.08, −0.36** **	**3.88**, **−0.24** *	#	1.07, 0.21
Cognition on discharge									
FIM score ≤ 5 on at least one area of cognition (dependency)	34	64	38	65	55	32	16	3	13
FIM score ≥ 6 on all areas of cognition (independent)	13	20	5	21	5	4	1	1	1
*X* ^2^, Phi	0.27, −0.05	**7.25, −0.26** *	**8.50, −0.34** *	**4.02, −0.20** *	**30.44, −0.53** **	**9.35, −0.29** *	#	#	#
Mobility on discharge									
FIM score ≤ 5 on at least one area of transfers/locomotion^a^	35	46	26	51	41	29	13	1	8
FIM score ≥ 6 on both transfers/locomotion^a^	12	38	17	35	19	7	4	3	6
*X* ^2^, Phi	**16.29, −0.39** **	0.89, −0.09	0.54, −0.09	**4.82, −0.21** *	**13.76, −0.36** **	**17.21, −0.40** **	2.00, −0.17	#	#

Abbreviations: CP, clinical psychology; DT, dietetics; MED, Medical; NS, nursing; NP, neuropsychology; OT, occupational therapy; PT, physiotherapy; SP, speech pathology; SW, social work.

^a^ Unable to fully deem these two groups exclusively independent or aspects of dependency due to the FIM score of ‘5’ having a dual score of ‘requires supervision’ AND/OR the ‘exception’ of ‘independence with household ambulation’.

* *p *≤ 0.05;

** *p *≤ 0.01; # invalid result due to low numbers.

Thirty‐three patients were discharged with an ‘independent’ level of cognition (FIM score 6 or 7 for FIM items 15–18). Those patients who were dependent on others for cognitive function (FIM score 5 or less) were more likely to have CGET documented than those who were independent, by an OT (*X* ^2^ = 7.25, *p* = 0.01), SP (*X* ^2^ = 8.5, *p* < 0.01), SW (*X* ^2^ = 4.02, *p* = 0.05), medical (*X* ^2^ = 30.4, *p* < 0.001) or nursing staff (*X* ^2^ = 9.35, *p* < 0.01). There were no significant differences between the dependent and independent cognition groups for PT.

### 3.1 Alignment to the Stroke Rehabilitation Guidelines

Figure 2 Caregiver Education and Training for primary rehabilitation provides the alignment to the Stroke Guidelines. Physiotherapy and OT were responsible for CGET related to Activity Limitations and Participation restrictions. SP documented CGET for communication and swallow‐related rehabilitation. Rehabilitation updates and overviews to caregivers was documented mainly by medical staff. Social work and OT documented most episodes of discharge and community integration (Figure 3 Caregiver Education and Training for Discharge) (SW *n* = 173; OT *n* = 176). There was consistency of input across the multidisciplinary team for family meetings, with Medical, OT and SW documenting most input.

**Figure 2:**
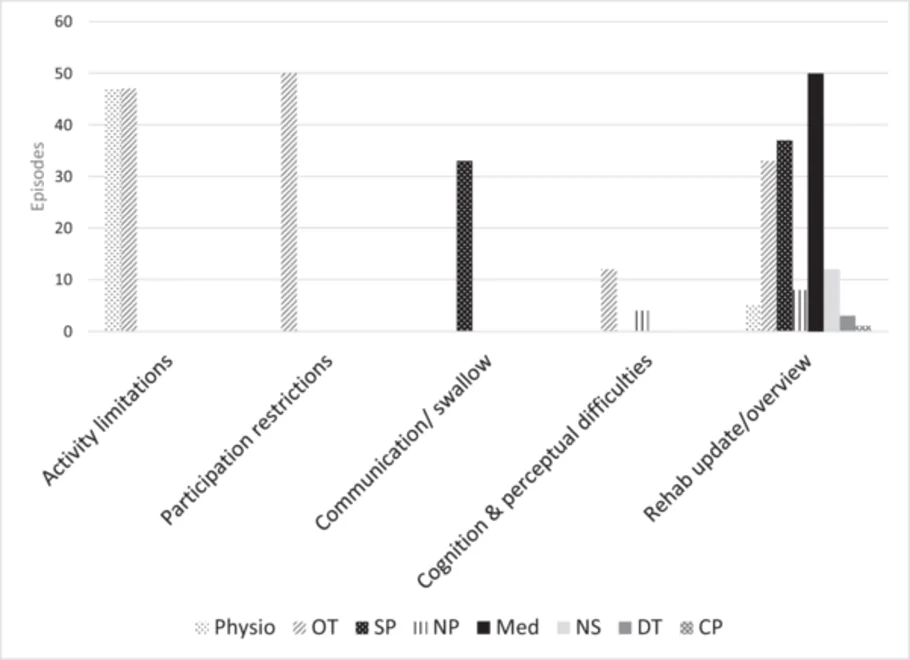
Documented counts of caregiver education and training for primary rehabilitation. *Physio = physiotherapy, CP = clinical psychology, DT = dietitian, NP = neuropsychology, NS = nursing staff, OT = occupational therapy, SP = speech pathology, SW = social work.

**Figure 3:**
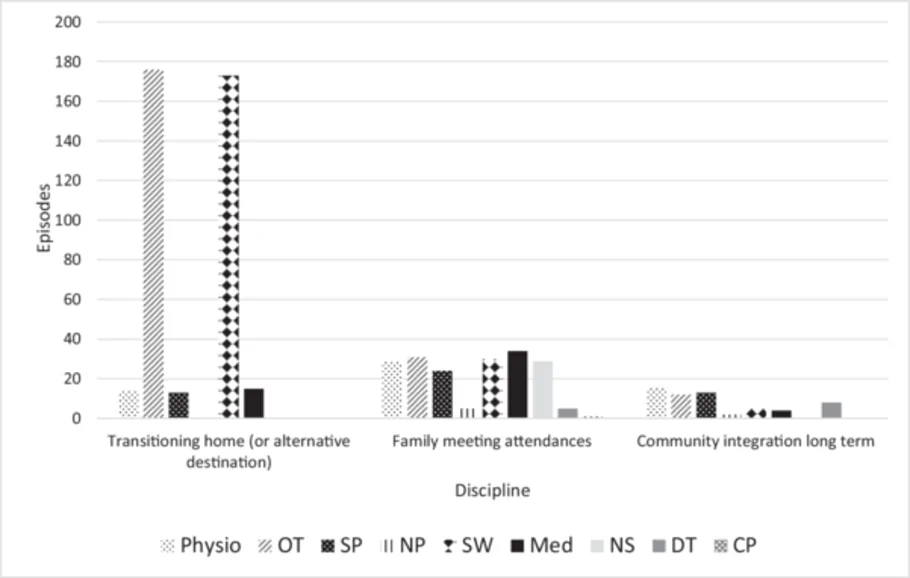
Documented counts of caregiver education and training for discharge. *Physio = physiotherapy, CP = clinical psychology, DT = dietitian, NP = neuropsychology, NS = nursing staff, OT = occupational therapy, SP = speech pathology, SW = social work.

## 4 Discussion and Conclusion

### 4.1 Discussion

This clinical audit investigated the current practice of CGET across the MDT on an inpatient stroke rehabilitation ward. The study shows that when caregivers were identified, education and training was not consistently documented. The OT and SW disciplines documented most CGET overall. For mobility, the literature indicates the subjective experience of caregivers is that they feel underprepared for mobility and transfers at home due to lack of ‘hands‐on’ training [7, 8]. In this audit, mobility education between OT and PT was documented well; however, physical training occurred less, even for patients requiring assistance to mobilise or transfer on discharge home.

The results for incorporating caregivers in therapy exercise programmes were low in this audit, whereas the evidence is clear that caregiver involvement can help improve treatment dosage and a positive trend in functional outcomes [23].

Pastore et al. [24] found that OTs preferred to provide ‘hands‐on’ training for CGET; however, the results in this study showed low rates for personal ADL training. This may have been due to the arrangement of community services for discharge, although these data were not captured specifically. However, with the frequent occurrence of other stroke sequalae such as incontinence, the additional CGET support is likely to have been helpful [25]. The lack of practical training provision is potentially due to time constraints that are a barrier to providing optimal care in the public health setting [24]. Nonetheless, practical training could be a future area of focus, particularly as caregivers often identify mobility and assistance with personal hygiene as the greatest challenges on discharge home [23, 25].

An estimated 40% of stroke survivors retain some cognitive impairment [26] which is associated with higher caregiver burden [27]. Cognitive changes from stoke is an under‐recognised area in stroke rehabilitation and is often de‐prioritised compared to physical rehabilitation by both clinicians and patients [26, 27]. In this study over one third of patients discharged home required assistance with memory; however, fewer than 11% of caregivers had documented evidence of CGET in relation to strategies to support memory loss. Caregivers need to be equipped with the skills and knowledge to cope with cognitive decline, particularly as the risk of dementia increases post‐stroke [28]. In this study, caregivers of patients with intact cognition were significantly less likely to have any CGET documented.

Caregiver supports on discharge are strongly recommended as part of Australian stroke guidelines [15], particularly to counter high rates of caregiver emotional distress and depression [29]. In this study there are opportunities to improve caregiver community support education, such as referral to support groups, online support programmes, counselling, financial information. Further, psychological support can provide management strategies for patient low mood or depression [30].

The roles of nursing staff during stroke rehabilitation are various and in addition to direct clinical care, incorporate education, support, advocacy and liaison with the multidisciplinary team [31]. This study showed some inconsistencies and low rates of CGET documented by nursing staff. For example, management of incontinence creates a large burden of care [25] yet there was minimal documentation of CGET for continence management strategies. As with any audit, provision cannot be proven without documentation, therefore documentation of CGET is a potential area to target to support caregivers [18].

Caregivers often report feeling overwhelmed during rehabilitation, finding it difficult to absorb important information [32]. This study showed that CGET was generally documented only once per domain. A single session can be challenging for caregivers to consolidate complex information while in an emotional state [32]. Future studies could triangulate the documented evidence with caregiver feedback on their experiences of CGET to measure effectiveness and direct future CGET accordingly. Repeated CGET were documented by SP for swallowing strategies and modified diet and fluids, reflecting the high risk associated with dysphagia and reinforcing CGET for safety reasons, and in line with recent recommendations to support patient and caregiver outcomes [33].

The Stroke Rehabilitation Guideline has a large focus on transitioning home. Given the evidence for caregivers feeling under‐prepared for discharge [6], the results of this study are positive for this aspect of CGET [34].

Complications post‐stroke contribute to hospital readmission rates for patients and cause significant distress in caregivers [35]. The documented MDT involvement in CGET for complications was less than other aspects of rehabilitation so this finding may suggest a gap in clinical practice that could be targeted in the future. Further, CGET for cognition changes was less than activity, participation limitations and communication difficulties and provides another opportunity to improve CGET during the rehabilitation phase to support global stroke recovery [13].

This study involved the MDT and in future research, a more focused audit on specific functional limitations may be more beneficial to assess CGET usefulness. Measuring both CGET frequency and caregiver outcomes would inform CGET effectiveness, particularly as a more recent systematic review showed no significant reduction to caregiver burden or stress following CGET [36]. Overall, the results of this study provide an opportunity to reflect on CGET practice in stroke rehabilitation and assist with identifying barriers and facilitators to optimising CGET post‐stroke.

Some limitations apply to this study. Only 5% of audited records were independently cross‐checked. Data were not collected for how many patients were discharged home with personal care services. Whilst all measures were taken to maintain the accuracy of coding, clinical audits have the complexities of differences in documentation practices and interpretation of clinical notes. A flaw in this study is the potential for clinicians not to document all instances and components of CGET, particularly regarding rehabilitation updates with caregivers. Whilst rehabilitation updates were included in the audit as a form of education, they were not necessarily reflected through documentation, especially with time pressures in the clinical setting. Further, any CGET provided before admission to the rehabilitation ward, e.g. on a comprehensive or acute stroke unit, would not have been captured.

Whilst the FIM is an excellent, person‐centred outcome measure for rehabilitation, it was a challenge to compare individual disciplinary input to discharge FIM domains due to the multidisciplinary team approach e.g. ‘eating’ limitations (FIM item 1) may have had input by SP, nursing and/or dietetics, thereby limiting the statistical analyses that could occur. Further, small subgroup sizes and multiple exploratory comparisons may limit the certainty of some subgroup findings. Also, without accurate data on pre‐morbid levels of function across all domains, it cannot be determined the exact functional level of baseline prior to each patient's stroke incident. Therefore, the documented CGET may have been biased relative to the stroke severity. Lastly, the codes were finalised during data collection prior to the decision to categorise the codes against the Stroke Rehabilitation Guidelines. The graphs representing the rehabilitation domains should be interpreted with caution due to the possible overlap of multiple domains and disciplines.

## 5 Conclusion

The clinical audit provided valuable insight into the current practice of documenting CGET by the MDT during rehabilitation post‐stroke. CGET can span numerous topics in preparation for discharge home; however, gaps were identified in the documentation of CGET for ADL, mobility and cognition. Further research is necessary to determine whether functional limitations of patients are being matched with the appropriate CGET.

### 5.1 Practice Implications

Multidisciplinary team documentation for stroke caregiver education and training should be standardised, particularly for cognition, continence, mobility and activities of daily living. Prospective evaluation that includes caregiver feedback may help determine whether documented education reflects the education and practical training received by caregivers, as well as its effectiveness.

## Funding

No funding was provided for this research, which was conducted as a component of a Master of Clinical Rehabilitation degree by the first author.

## Ethics Statement

This project was approved by Barwon Health Human Research Ethics Committee (21/177).

## Conflicts of Interest

The authors declare no conflicts of interest.

## Data Availability

Data is available on request from the authors.
